# **Taking the Relationship Between Populism and Healthcare Seriously: A Call for Empirical Analysis Rather Than Moral Condemnation** Comment on "A Scoping Review of Populist Radical Right Parties’ Influence on Welfare Policy and its Implications for Population Health in Europe"

**DOI:** 10.34172/ijhpm.2020.180

**Published:** 2020-09-23

**Authors:** Martijn Felder, Iris Wallenburg, Syb Kuijper, Roland Bal

**Affiliations:** Health Care Governance, Erasmus School of Health Policy & Management, Erasmus University, Rotterdam, The Netherlands.

**Keywords:** Populism, Healthcare, Welfare States

## Abstract

In this commentary, we reflect on Rinaldi and Bekker’s scoping review of the literature on populist radical right (PRR) parties and welfare policies. We argue that their review provides political scientists and healthcare scholars with a firm basis to further explore the relationships between populism and welfare policies in different political systems. In line with the authors, we furthermore (re)emphasize the need for additional empirical inquiries into the relationship between populism and healthcare. But instead of expanding the research agenda suggested – for instance by adding categories or niches in which this relationship can be observed – we would like to challenge some of the premises of the studies conducted and reviewed thus far. We do so by identifying two concerns and by illustrating these concerns with two examples from the Netherlands.

## Introduction

In light of adverse public healthcare trends – such as declining life expectancies and increased health inequalities – Rinaldi and Bekker^1^ have examined the relationship between populist radical right (PRR) parties and welfare policy reforms. In order to do so, the authors identified and analyzed existing literature on PRR parties, political systems and welfare policies in Europe. Due to the limited number of – and variety in – literature available, they conducted a scoping review.

Studying the effects of political parties on welfare policies and their outcomes is not new. Nevertheless, such studies have traditionally focused on conventional political parties that could be placed on a left-right continuum. These studies suggested that left-winged parties are generally responsible for the expansion of welfare policies, whereas right-winged parties are responsible for the retrenchment of such policies.^2^ Less work has however been done on the effects of PRR parties on welfare policies. This is surprising, particularly so given the fact that PRR parties (and many conventional parties alike) do not easily fit into a left-right political continuum. In fact, these parties have come to position themselves in very different terms, such as in juxtaposition of a corrupt elite.^3^ By placing the relationship between PRR parties and welfare policies on the scientific agenda, Rinaldi and Bekker have thus made an important contribution to the political sciences.

 Next to its timeliness and urgency, Rinaldi and Bekker’s scoping review is also well-structured and written. It provides us with a clear methodological description on the selection of papers included, the analytical steps taken and interpretations made. Based on their analysis, the authors conclude that: (I) the literature examined suggests that PRR’s welfare chauvinism is harmful for welfare policies in general and public health policies more specifically; (II) that the make-up of different political systems (eg, constitution; multiparty government; corporatist tradition; EU law) possibly mediates the risks of PRR ideology; and (III) that PRR parties affect welfare policy differently in different systems. These are conclusions that beg for more empirical research into the intricacies of – and political dynamics in – these different systems.

## An Important Research Agenda

Rinaldi and Bekker’s scoping review provides political scientists and healthcare scholars with a firm basis to further explore the relationships between populism and welfare policies in different political systems. This is evidenced in the commentaries which – at the time of writing this commentary – have already been published in response to their scoping review. McKee and colleagues^4^ for instance suggest studying the relationship between populist leaders and the spread of coronavirus disease 2019 (COVID-19); Moise^2^ suggests expanding the study into eastern-European countries; and Stronks and Agyemang^5^ call for interdisciplinary work between healthcare scholars and political scientists and to study the relationship between populism and healthcare from a systems perspective.

 We too would like to take the opportunity to stress the need for additional empirical inquiries into the relationship between populism and healthcare. But instead of expanding the suggested research agenda – for instance by adding categories or niches in which this relationship can be observed – we would like to challenge some of the premises of the studies conducted so far.

## Some Concerns About the Direction of This Research Agenda

Our main concerns revolve around the reductions and inferences made with reference to populism (see also De Cleen and Speed,^3^ who raise concerns that resonate with our own). In the scoping review, the literature behind it, as well as the commentaries that followed, populism is defined as *thin ideology*, based on the principles of *antiestablishment*, *nativism* and *authoritarianism*. As such, it is conceived of as different from and threatening to the established political order and the welfare state. Populism is the danger from the extreme right against which naïve citizens, minority groups and deliberative and consensus based society need to be protected.^6^ A cause in which the sciences can play their part.

The above-mentioned approach produces normative (research) questions, such as: *how does populism influence democratic quality*?^7^ or: *denialism, what is it and how should scientists respond?*^8^ Moreover, it produces scientific texts that are riddled with moralizing passages, such as when referring to *harmful beliefs* and *fake experts.*^1,8^ Through such questions and texts, a specific epistemological (but also political-ideological) scientific community is established. One that is based on (liberal) political theories and hypotheses rather than critical and empirical scrutiny of populist movements and their expressions. In doing so, however, political scientists and healthcare scholars run the risk of distancing themselves from the very political movements and societal developments they seek to understand. To counter this development, we would like to raise two points for discussion. We do so in the next four paragraphs.

###  The First Concern – PRR Parties and the Welfare State

Our first point concerns the suggested relationship between PRR parties and welfare policies. By studying the effects of PRR parties on welfare policies, Rinaldi and Bekker^1^ produce a hypothetical link between these parties and the breaking down of the welfare state. There are, however, authors that turn this line of reasoning on its head. Chantal Mouffe,^9^ for instance, relates very differently to PRR parties. According to her, populism has emerged in response to trends in Western States in which conventional political parties – left and right – started to find one another at the center. This has had three consequences: (I) voters do no longer feel represented by their consensus seeking political representatives; (II) a neoliberal policy agenda emerged characterized by the introduction of market mechanisms; (III) such neoliberal policy agendas have led to new uncertainties, stemming from a retrenchment of welfare policies and (misplaced) devotion to the regulatory qualities of markets.^10^ These developments pushed apprehensive citizens to find new forms of political representation.

It is not our intention to take sides here. Nevertheless, the above-mentioned suggests that the breaking down of the welfare state is tied to conventional party politics and the mechanisms of (consensus based) democracy, rather than the emergence of PRR parties. In fact, it even suggests that the emergence of PRR parties can be explained in response to – instead of as causing – the breaking down of the welfare state. Our point is that (political) cause(s) and (healthcare policy) effects seem to be more intricately folded than the current research agenda suggests. As political scientists and healthcare scholars, we should therefore avoid participating in a blame game in which we can find comfort in the fact that adverse healthcare trends are caused by the morally condemned and ignorant populists. If we are to learn something from Mouffe,^9^ that is exactly how some conventional political parties have managed to avoid taking responsibility for the consequence of their own neoliberal policy agendas.

###  The Second Concern – What Is Populism?

Our second point concerns the conceptualization of populism in general and PRR more specifically. Studying the relationship between populism and welfare policies by focusing on PRR parties is a practical and analytical choice. Nevertheless – and as Rinaldi and Bekker^1^ suggest themselves by referring to the Trump example – it also means that all kinds of populist manifestations are ignored. Although we understand the choice the authors made in light of their scoping review, we also argue that the research agenda that is subsequently proposed needs to open-up to different expressions of populist movements. We suggest to start by rephrasing – in analytical terms – what populism actually is.^3^ In order to do so, we return to scholarship based on the work of Chantal Mouffe and Ernesto Laclau.

According to Demir,^11^ who insightfully combines the work of Mouffe and Laclau: ‘ *populism is not a failure or a malfunction of the democratic order, nor is it the pathology of capitalism. It is not an ideology, and it does not have a specific program. It is not a political regime, either. Instead, it is a way of making politics that is compatible with various political structures and can take different ideological forms according to time and space.’ *It is about building a political frontier that discursively divides society into camps: the common people versus the corrupt elite. Moreover, it seeks to find new ways of political representation where people from different walks of life feel their voices are no longer heard.^12^ In this light, divisions can be organized on the basis of different topics (hence the so-called absence of one ideology) and they can take on many different forms (far beyond PRR party political representation). Different groups can come together at different times, for different reasons and in juxtaposition of different others (eg, Gilets Jaunes). Our point is that, if we want to take democracy seriously, such movements and the way they emerge, develop and dissolve (only to reappear somewhere else), deserve close empirical scrutiny rather than moral condemnation.

## Two Examples

Not only does the above-mentioned make populism unpredictable (as opposed to the somewhat predictable moves of PRR parties who need to stick to capital P political conduct), it also means that the effects of populism on healthcare are more diffuse than suggested in the literature reviewed.^13^ We would like to illustrate this point with two examples from the Netherlands.

###  The First Example – Rallying Nurses

In the summer of 2019, a new bill was proposed for Dutch healthcare. The bill decreed that a formal distinction should be made between bachelor trained and vocationally trained nurses; in doing so closing a long lasting issue in the organization and valuation of Dutch nursing work (the Netherlands differs from most other countries in not differentiating between different levels of nursing work). By making this distinction, the bill aimed to make the nursing profession more attractive, especially for higher educated nurses. The bill had been developed by an independent (expert) commission and with the support of the biggest nursing association. However, soon after the bill was presented to a wider audience, a small but outspoken group of nurses started rallying against it, fearing that the bill would have consequences for their position and everyday work. They positioned themselves in terms of representing all nurses (the people) and in juxtaposition to a corrupt elite (the expert commission and the professional organization). Meanwhile, they managed to get extensive media coverage. Their attacks on the new bill were so fierce – and alternative voices from the nursing community were so scarce (and hardly paid attention to in the media) – that the Minister of Healthcare renounced the bill’s introduction. Meanwhile the board of the nursing association was forced to step down. A relatively small group of rallying nurses thus managed to oppose policy reforms without (*a*) any insight into the extent to which they actually represented the Dutch nursing community; and (*b*) through channels other than established political institutions. Our point with this first example is that populism (and the different ways in which opposition is mobilized) affects healthcare beyond the mechanisms of party politics.

###  The Second Example – New Funds for Elderly Care

In 2016, Hugo Borst (writer and public figure) and Carin Gaemers (sociologist and researcher) published a manifesto titled ‘Focus on Elderly Care’ (translated from Dutch). The manifesto demanded that the Dutch government should take responsibility for – and improve – healthcare provided to vulnerable elderly in nursing homes. The manifesto claimed that nursing home residents lived monotonous lives due to lack of personal attention and lack of healthcare quality. This was, the manifesto continued, in direct violation of the social rights of the elderly. The manifesto gained unprecedented publicity. Because of that, the manifesto’s authors managed to take the discussion out of parliament (where the topic had been discussed on and off) and into the public domain. There, they built a political frontier between a large group of concerned citizens and a small group of bickering politicians. Individual politicians were subsequently forced to take political responsibility for the described situation in the elderly homes. Soon after, Fleur Agema of the Party for Freedom (generally considered a PRR party) introduced a resolution in the Dutch parliament which was supported by all other parties (from the left and right). The parties unanimously agreed to make more funds available to improve the quality of elderly care. Our point with this second example is that populism does not only assert itself as a movement against welfare. At times, it also manages to protect and re-establish welfare policies that seem to disappear in favor of market regulation and austerity. It does so by making use of both conventional (and vertical) political institutions as well as unconventional forms of (more horizontal) political organization. Also in the scoping review of Rinaldi and Bekker and the commentaries that followed, such positive exceptions are mentioned.^3^

## Conclusion

The work of Carl Schmitt – a 20th century scholar who has fallen out of grace due to his association with the Nazi regime – has recently been (re)discovered by scholars that seek to understand a revived politics of ‘us’ versus ‘them’ and the spatial, social and juridical consequences this has.^8,14,15^ Schmitt already warned in the 1950s that a proliferation of new (and rather hierarchical) institutions in search for a united Europe, would lead to chaotic and ad hoc forms of partisan (counter) organizations on the ground. This is not necessarily something to look forward to, as it could lead to all kinds of invisible and unpredictable exclusions, in different places and against different kinds of others.^5^

In this light, studying the relationship between populism and healthcare is of major importance. On this we fully agree with Rinaldi and Bekker and all other the scholars that have taken the opportunity to comment on their work. But such studies should move beyond equating populism to PRR parties^3^ and/or the effects that some archetypical PRR parties and leaders have had on capital P politics and welfare policies.^4^ In addition (or maybe even instead), we should study how in the context of: (*a*) disintegrating welfare states and the insecurities that come with it; and (*b*) a lack of trust in (established) political representation to discuss and deal with such insecurities; different kinds of populist movements and expressions emerge and start influencing healthcare decision-making in unpredictable ways.

## Ethical issues

 Not applicable.

## Competing interests

 Authors declare that they have no competing interests.

## Authors’ contributions

 MF wrote a first draft and IW, SK, and RB contributed with feedback, examples and revisions.
